# Interpretable multimodal fusion for skin lesion classification using dermoscopic images and patient metadata

**DOI:** 10.3389/fmed.2026.1871689

**Published:** 2026-07-29

**Authors:** Emrullah Sonuç, Qusay Saihood, Yusuf Yargı Baydilli, Ender Özcan

**Affiliations:** 1 Computational Optimisation and Learning Lab, School of Computer Science, University of Nottingham, Nottingham, United Kingdom; 2 Department of Computer Engineering, Karabuk University, Karabük, Türkiye; 3 Department of Computer Engineering, Hakkari University, Hakkari, Türkiye

**Keywords:** Bayesian optimization, clinical decision support, computer-aided diagnosis, dermoscopic imaging, interpretable AI, multimodal data fusion, skin lesion classification, vision transformer

## Abstract

**Introduction:**

Accurate and early classification of skin lesions is essential for the early detection of disease and improved clinical outcomes. However, automated multiclass classification is still hindered by severe class imbalance, high inter-class visual similarity, and the comparatively under-explored use of complementary clinical metadata relative to image-only pipelines, despite growing interest in such metadata fusion. In line with the growing demand for artificial intelligence (AI) tools that integrate heterogeneous data in clinical scenarios, this study presents a multimodal machine learning-based computer-aided diagnosis (ML-CAD) framework that fuses dermoscopic images with patient metadata.

**Methods:**

The framework follows a five-phase pipeline. A structured multimodal balancing strategy combines class-wise Synthetic Minority Oversampling Technique for Nominal and Continuous features (SMOTENC) for metadata with controlled image augmentation. This is followed by cross-modal alignment to preserve clinical consistency. A fine-tuned Vision Transformer (ViT-B/16) at reduced input resolution performs feature extraction and late fusion with encoded metadata, yielding an 800-dimensional multimodal representation. Individual and stacked ensemble classifiers are subsequently trained on the fused features, with Bayesian optimization based on Gaussian process surrogates used for hyperparameter tuning. Gradient-weighted Class Activation Mapping (Grad-CAM) provides a visual interpretation across all seven lesion categories.

**Results:**

Experiments on the HAM10000 dataset demonstrate that the proposed SVM+KNN stacking ensemble achieved an accuracy of 98.55% and a ROC-AUC of 99.88%, achieving competitive accuracy among recently reported methods under broadly similar balanced settings.

**Discussion:**

The findings underscore the necessity of an AI framework that integrates imaging and clinical data to provide interpretable clinical decision support. This framework represents a step toward providing clinicians with multimodal, interpretable decision support. We emphasize, however, that external and prospective clinical validation remain necessary before translation into routine dermatological practice.

## 1 Introduction

The integration of artificial intelligence (AI) with multimodal medical data has emerged as a transformative paradigm in modern healthcare, offering opportunities to enhance diagnostic accuracy and clinical decision-making by jointly leveraging imaging, textual records, and patient-level clinical information (1). Among the clinical domains expected to benefit most from such advances, dermatology stands out, as skin cancer is among the most prevalent forms of cancer worldwide, with 1.20 million new cases reported in 2022 according to the World Health Organization.^1^ The incidence continues to rise globally, driven by population aging and increased ultraviolet (UV) exposure (2, 3). A skin lesion is defined as any pathological change in the skin, ranging from benign conditions such as moles to malignant forms such as melanoma. Early diagnosis is crucial, as it can improve the 5-year survival rate to over 99% (4). However, accurate diagnosis is challenging due to confounding factors including surface reflections, color variations, and the presence of hair or moles (4). While dermoscopy improves diagnostic accuracy from approximately 60% to 75%, clinical examination often remains inconclusive, requiring invasive and costly biopsies (5, 6).

These limitations have motivated the development of machine learning-based computer-aided diagnosis (ML-CAD) systems that can integrate dermoscopic imaging with clinical context. Deep learning techniques, particularly Convolutional Neural Networks (CNNs), have advanced skin lesion analysis, but are limited in capturing global contextual dependencies. This has motivated research into Vision Transformers (ViTs), which model long-range dependencies through self-attention mechanisms (7, 8). Despite these advances, several challenges persist that hinder clinical translation. Most studies focus on binary classification rather than clinically relevant multiclass categorization (9, 10). Data augmentation is typically treated as supplementary rather than integral (11, 12). Furthermore, the fusion of clinical metadata (age, sex, and lesion localization) remains comparatively under-explored relative to image-only pipelines, although interest is growing rapidly, and few studies address systematic hyperparameter optimization or model interpretability (13). These gaps reflect a broader challenge in clinical AI: combining and interpreting heterogeneous medical data in a way that is both accurate and trustworthy in real practice.

This study proposes an enhanced multimodal framework for seven-class skin lesion classification on the HAM10000 dataset, contributing to the broader goal of advancing AI-driven multimodal data fusion as a potential basis for future clinical decision support, subject to the external and prospective validation discussed in Section 5. The key contributions are:

- A multimodal balancing strategy combining Synthetic Minority Oversampling Technique for Nominal and Continuous features (SMOTENC) for metadata with controlled image augmentation and cross-modal alignment to address class imbalance.
- A ViT-based feature extractor with reduced input resolution for computational efficiency, integrated with clinical metadata through late fusion.
- Systematic evaluation of individual and ensemble classifiers on multimodal features, with Bayesian optimization for hyperparameter tuning.
- Grad-CAM-based visual interpretation of both correctly classified and misclassified samples across all seven lesion categories, providing visual interpretability of the model's decisions rather than a complete explainability suite.

The remainder of the manuscript is organized as follows. Section 2 reviews related work. Section 3 details the proposed materials and methods. Section 4 reports the experimental results. Section 5 discusses the findings, comparisons with the literature, and limitations. Section 6 concludes the work and outlines future directions.

## 2 Related work

Given the importance of early classification of skin lesions, which is crucial for reducing complications and improving cure rates, ML has emerged as a powerful tool capable of analyzing lesion images and identifying hidden patterns for early diagnosis. Over the past few years, numerous studies have investigated various ML techniques for skin lesion classification, ranging from traditional methods based on manually extracted features, to CNN-based approaches that have revolutionized medical image analysis, as well as ensemble learning (EL) strategies designed to combine multiple models into powerful frameworks (1).

Bechelli and Delhommelle (9) conducted a comprehensive study to explore the capabilities of a wide range of ML techniques, including Decision Tree (DT), Naive Bayes (NB), Logistic Regression (LR), Linear Discriminant Analysis (LDA), K-Nearest Neighbor (KNN), customized CNN, Xception, VGG16, ResNet50, as well as EL models, for skin cancer classification. Similarly, Shetty et al. (14) also explored both traditional ML and CNN approaches, expanding the task to include multiclass classification. Ali et al. (15) evaluated eight versions of the EfficientNet model (B0–B7) for multiclass skin lesion classification to determine the most efficient version for this task. Their results indicated that EfficientNet-B4 outperformed other versions, demonstrating that greater model complexity does not necessarily yield better performance.

Several studies have addressed data imbalance, a major challenge that significantly impacts model accuracy. Velaga et al. (12) proposed an intelligent diagnosis system using the Synthetic Minority Over-sampling Technique (SMOTE) method to balance the data and enhance classification accuracy. Govindu et al. (16) explored the combined use of SMOTE, oversampling, and undersampling to improve data balance and classification performance. Tahir et al. (11) combined SMOTE-Tomek with a customized CNN model to classify four types of skin cancer more accurately. Alwakid et al. (17) addressed data imbalance using image data augmentation to generate additional synthetic images. Furthermore, Abbas and Elbehiery (18) addressed data imbalance by employing geometric data augmentation techniques, including translations, rotations, and zooming, to generate additional training samples for four CNN-based models. Conversely, Asaduzzaman et al. (19) used a Generative Adversarial Network (GAN) discriminator to filter out fake images and applied SMOTE for class balancing.

Another research direction involves hybrid and EL models to enhance skin lesion classification accuracy. Khan et al. (10) proposed a hybrid model combining VGG19 and Network-In-Network (NIN) architectures for binary classification. In multiclass classification, Khan et al. (20) proposed a DenseNet-CNN hybrid model to improve classification accuracy. In an EL-based approach, Khan et al. (21) built a model combining three fine-tuned models to improve the accuracy of classifying seven types of skin lesions. Natha et al. (22) aimed to improve the performance of EL models by using a genetic algorithm for feature selection.

Although CNN-based models perform well in capturing local spatial features, they are limited in capturing global contextual dependencies. As a result, research interest has shifted toward transformer-based models, inspired by their remarkable success in natural language processing (NLP). Transformer-based models can be more efficient in handling changes in image scale and orientation because their attention mechanisms capture global dependencies more effectively than CNN-based models. Accordingly, Aladhadh et al. (23) developed the Medical ViT (MViT) for skin lesion classification. The MViT segments lesion images into patches and feeds them sequentially to a transformer encoder, similar to word embedding. A Multilayer Perceptron (MLP) then classifies the seven lesion types. Xin et al. (24) extended the original ViT by introducing overlapping multi-scale patch embeddings to improve fine-scale feature capture and using contrastive learning to enhance feature discrimination. Similarly, Yang et al. (7) proposed a four-phase ViT framework consisting of class rebalancing, patch generation and flattening into tokens, transformer encoding, and final classification, achieving accuracies of 94.10% and 80.50% on HAM10000 and DERMOFIT, respectively. Furthermore, Himel et al. (8) explored six ViT-based models to diagnose whether a skin lesion is malignant or benign. While Rao et al. (25) attempted to improve multiclass ViT classification using a ViT-based GAN to address data imbalance, the results suggest overfitting, limited generalization, and high computational cost.

Recent studies have also emphasized the value of incorporating patient metadata into skin lesion classification models. Tang et al. (26) proposed a two-stage multimodal framework that combines clinical images, dermoscopic images, and patient metadata for multi-label skin lesion classification. Similarly, Khurshid et al. (27) introduced a dual-stage feature refinement model that integrates lesion images with clinical and demographic metadata to improve robust multiclass classification. Saeed et al. (28) further demonstrated that combining dermoscopic images with patient attributes through an adaptive weighted ensemble improves performance compared with image-only models. More recently, Tran-Van and Le (29) proposed a cross-attention-based fusion strategy to integrate dermoscopic images and metadata, achieving strong performance on HAM10000. These studies suggest that patient metadata provides complementary information that can support skin lesion classification when combined with image features.

Despite the progress reported in previous studies, several important challenges remain. Most studies have used SMOTE or GAN-based approaches to address class imbalance. SMOTE is typically used to balance tabular data rather than high-dimensional image data, whereas GAN-based models can produce high-quality synthetic images but are computationally expensive. Another key limitation is the limited utilization of metadata associated with skin lesion images. Although clinical characteristics such as age, sex, and lesion location are clinically important, most studies rely solely on image features, overlooking information that could improve classification, particularly for similar lesion types. Moreover, many previous studies have paid limited attention to systematic hyperparameter tuning, often relying on manual tuning. In addition, few studies address model interpretability, prioritizing accuracy over explainability and limiting clinical transparency and real-world adoption (13). In fact, the large majority of prior HAM10000 studies report no interpretability analysis at all; where interpretability is considered, it is typically restricted to a single visualization technique. Accordingly, the present work does not claim a comprehensive explainability framework, but rather provides visual interpretability through Grad-CAM, which still goes beyond the purely accuracy-oriented reporting of most existing approaches. Overall, studies that achieved high classification accuracy focused primarily on binary classification, while studies that extended to multiclass classification still require further improvement to achieve reliable multiclas performance.

## 3 Materials and methods

**Figure 1:**
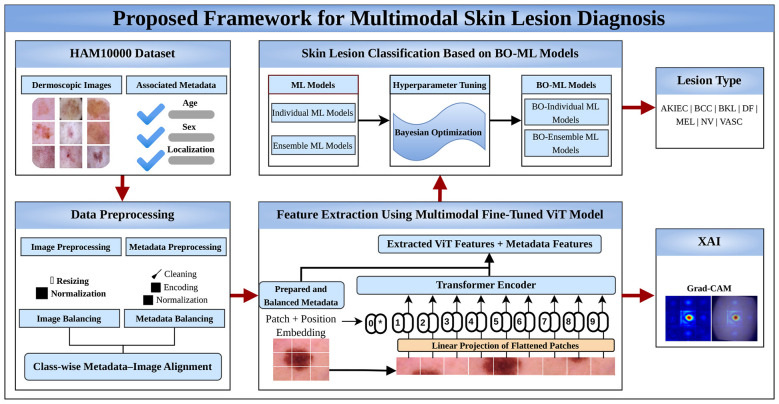
Schematic of the proposed methodology: data preprocessing with multimodal balancing, ViT-based feature extraction with late fusion, Bayesian-optimized ML classifiers, and visual interpretation.

The proposed framework comprises five phases: (1) data preprocessing with multimodal balancing, (2) ViT-based feature extraction with metadata fusion, (3) individual and ensemble classification, (4) Bayesian hyperparameter optimization, and (5) visual interpretation. Figure 1 illustrates the overall framework.

### 3.1 Dataset description

**Figure 2:**
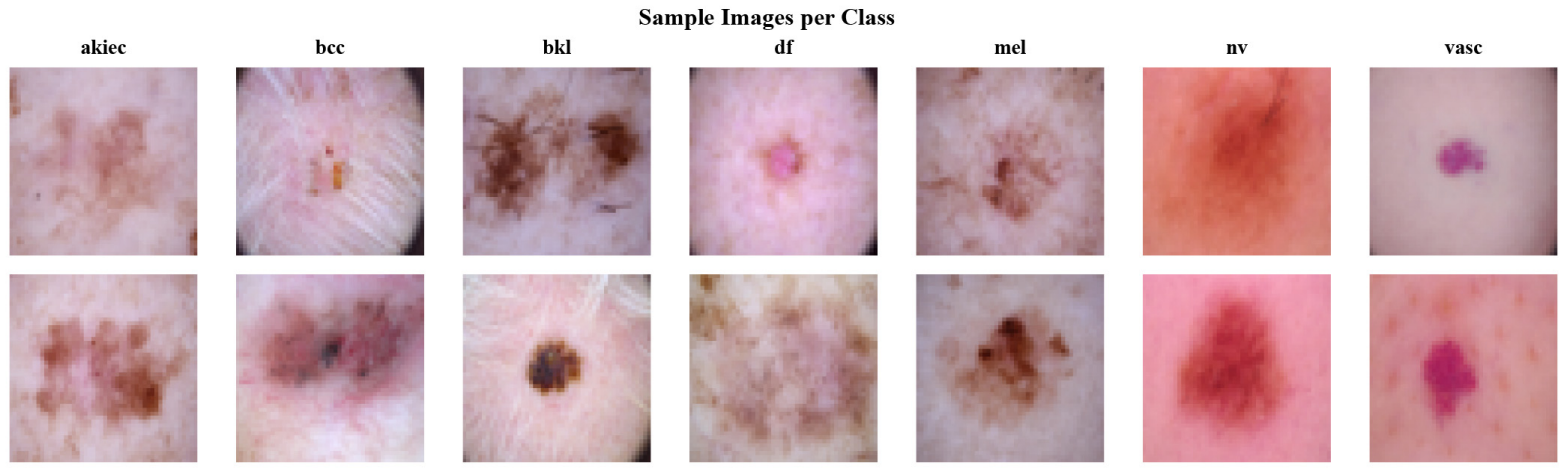
Representative sample images from each skin lesion class in the HAM10000 dataset.

**Figure 3:**
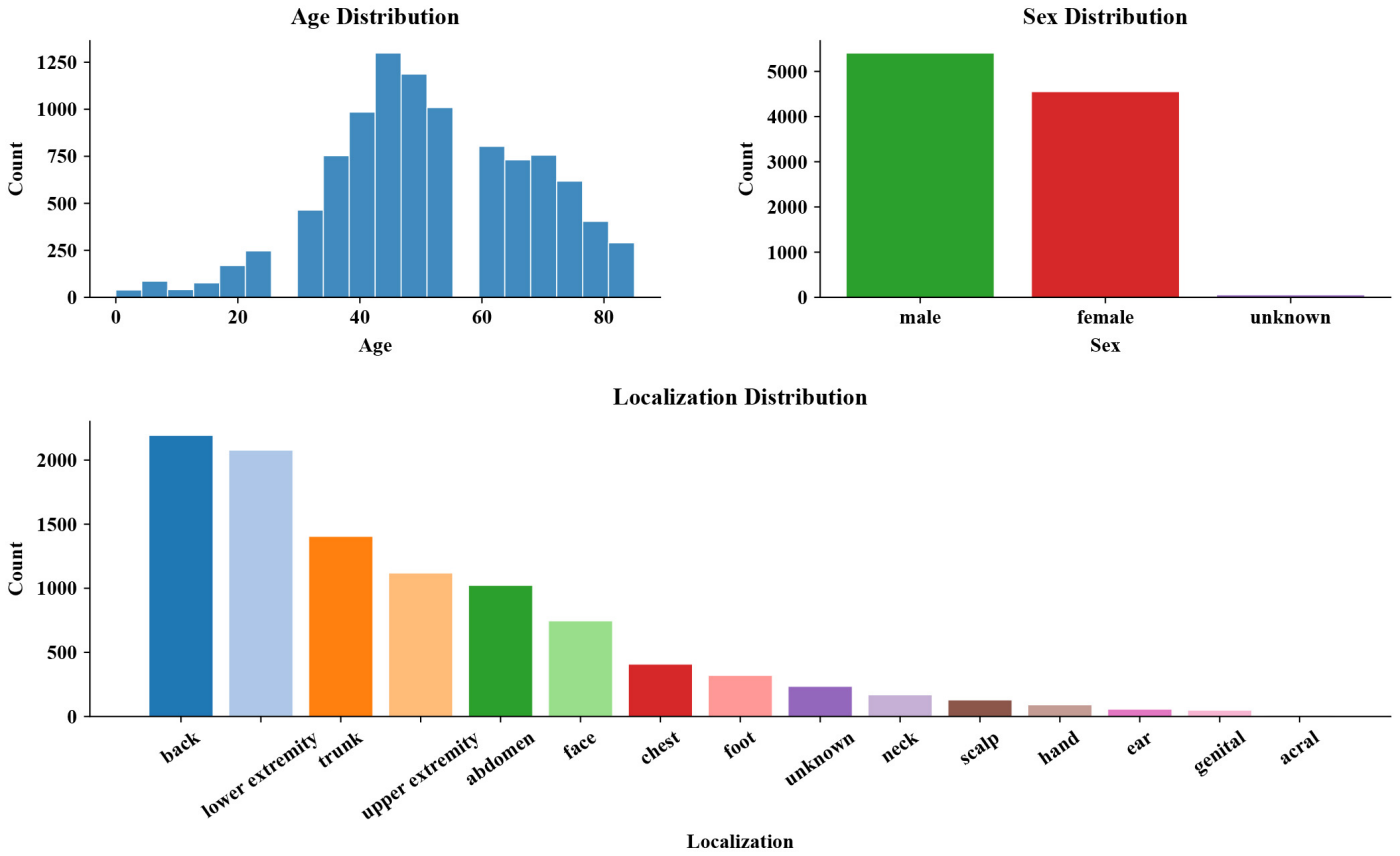
Distribution of patient age, sex, and lesion localization in the HAM10000 metadata.

The HAM10000 dataset (30) contains 10,015 dermoscopic images of seven pigmented skin lesion types: actinic keratoses (AKIEC), basal cell carcinoma (BCC), benign keratosis-like lesions (BKL), dermatofibroma (DF), melanoma (MEL), melanocytic nevus (NV), and vascular lesions (VASC). Images are RGB JPEG files at approximately 600 × 450 pixels. Approximately 50% were confirmed by histopathology, with the remainder diagnosed through expert consensus or confocal microscopy. The dataset includes clinical metadata: patient age, sex, and lesion localization. Figure 2 shows representative samples, and Figure 3 illustrates metadata distributions.

### 3.2 Data preprocessing

#### 3.2.1 Metadata preprocessing

Missing values in the age feature were replaced with the median. Sex was encoded using binary label encoding (0/1). Lesion localization and type were one-hot encoded. The processed features (age, sex, and localization) were combined into a 17-dimensional metadata vector.

#### 3.2.2 Image preprocessing

Images were resized to 48 × 48 pixels for uniform dimensions and compatibility with the model input. Pixel values were normalized to [0, 1] by dividing by 255.0 to improve convergence and training stability.

#### 3.2.3 Multimodal data balancing strategy

The HAM10000 dataset exhibits severe class imbalance (Table 1), with NV containing 6,705 samples and DF only 115. To address this, a structured multimodal balancing strategy was applied.

**Table 1:** Class distribution before and after data balancing.

Class	Before balancing	Train	Validation	Test	After balancing
NV	6,705	4,694	1,006	1,005	6,705
MEL	1,113	779	167	167	6,705
BKL	1,099	769	165	165	6,705
BCC	514	360	77	77	6,705
AKIEC	327	229	49	49	6,705
VASC	142	99	21	22	6,705
DF	115	80	17	18	6,705
Total	10,015	7,010	1,502	1,503	46,935

The original data were stratified into 70%/15%/15% training, validation, and test partitions at the lesion-ID level prior to any augmentation. Balancing was applied to the training partition only, whereas the validation and held-out test partitions retain the original (imbalanced) HAM10000 class distribution, so the reported metrics reflect performance under a clinically realistic class prevalence. The “After Balancing” column refers to the balanced training partition.

##### 3.2.3.1 Metadata balancing

SMOTENC (31, 32) was applied independently within each minority class to generate synthetic metadata vectors. Unlike standard SMOTE, SMOTENC handles mixed continuous (age) and categorical (sex, localization) features, preserving clinical plausibility. Class-wise application prevents cross-class contamination.

##### 3.2.3.2 Image balancing

**Figure 4:**
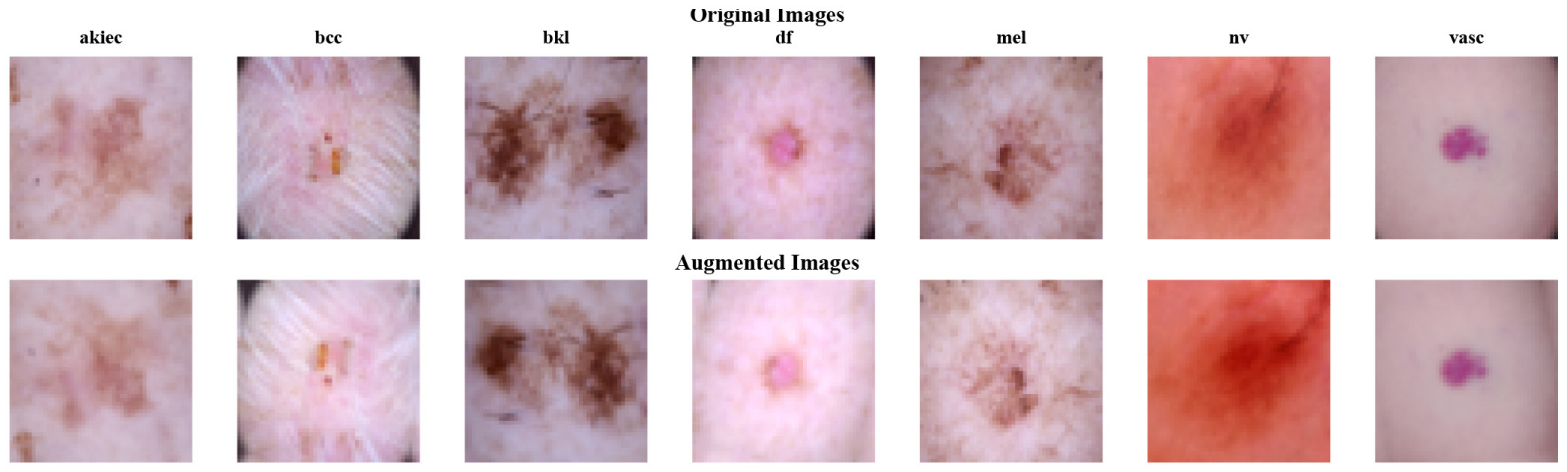
Examples of original and augmented skin lesion images.

Albumentations (33) was used to generate additional images for minority classes through controlled transformations: horizontal and vertical flips (50% probability each), random rotation (±30°, 70% probability), and brightness/contrast adjustments (Figure 4). Each class was balanced to 6,705 samples.

##### 3.2.3.3 Cross-modal alignment

To prevent data leakage, the original dataset was first split at the lesion-ID level prior to any augmentation. Subsequently, SMOTENC oversampling and image augmentation were applied in a controlled manner following the data partitioning strategy. For original samples, each image was paired with its associated metadata. For synthetically generated metadata, an image was randomly selected from the same class and augmented to construct a corresponding input, preserving class-level semantic coherence. All synthetic metadata vectors generated by SMOTENC were paired exclusively with same-class images drawn from within the same training partition; because the lesion-ID-level split precedes augmentation, no synthetic pairing crosses patient boundaries or leaks into the evaluation data.

### 3.3 Feature extraction and multimodal fusion

A modified ViT-B/16 architecture (34) was employed for feature extraction. The model was initialized with ImageNet pre-trained weights, and the last ten transformer encoder layers were unfrozen for task-specific fine-tuning.

The spatial resolution was reduced from the conventional 224 × 224 to 48 × 48 × 3. Under this configuration with patch size *P* = 16, the token sequence length is *N* = 48^2^/16^2^ = 9, compared to *N*_224_ = 196 at standard resolution. Since self-attention scales as $O\left(N^{2}\right)$, this yields an approximately 474-fold reduction in self-attention operations (196^2^/9^2^≈474), substantially lowering computational cost while retaining global modeling capability. The reduced resolution was validated empirically: classification performance on the fused features remained competitive despite the lower spatial detail, as the downstream ML classifiers operate on the 800-dimensional feature space rather than raw pixels.

A late fusion strategy integrates clinical metadata. The ViT backbone produces a 768-dimensional embedding, normalized via batch normalization. The 17-dimensional metadata vector is processed through a fully connected layer (32 units, ReLU activation) with batch normalization. The normalized image embedding (768-d) and metadata representation (32-d) are concatenated to form an 800-dimensional multimodal vector. This is fed through a dense layer (128 units, GELU), dropout (0.3), batch normalization, and a Softmax classification layer. The complete configuration is summarized in Table 2.

**Table 2:** Configuration of the ViT-based multimodal framework.

Category	Parameter	Value
Backbone	Model	ViT-B/16
	Input size	48 × 48 × 3
	Patch size	16
	Transformer layers	12
	Attention heads	12
	Embedding dimension	768
	Fine-tuned layers	Last 10
Training	Optimizer	Adam
	Batch size	64
	Initial learning rate	1 × 10^−4^
	Epochs	50
	LR scheduler	ReduceLROnPlateau
	LR reduction factor	0.5
	Min learning rate	1 × 10^−7^
	Early stopping patience	5
Regularization	Dropout	0.3
Loss	Focal α	0.25
	Focal γ	2.0
Fusion	Metadata dimension	17
	Metadata FC units	32
	Fusion dimension	800
	Fusion dense units	128
	Activation function	GELU

### 3.4 Skin lesion classification

Classification was performed in two scenarios using the fused multimodal features as input.

#### 3.4.1 Individual ML models

Four classifiers were evaluated: Support Vector Machine (SVM) (35), Decision Tree (DT) (36), K-Nearest Neighbors (KNN) (37), and Logistic Regression (LR) (38). SVM was selected for its effectiveness in high-dimensional feature spaces, KNN for local neighborhood-based decision making, DT for its interpretable rule-based structure, and LR for probabilistic classification with efficient training.

#### 3.4.2 Ensemble learning methods

Three ensemble strategies were employed (39): (1) *Bagging*: Random Forest (RF) (40) and Extremely Randomized Trees (ET) (41); (2) *Boosting*: Extreme Gradient Boosting (XGB) (42) and Light Gradient Boosting Machine (LGBM) (43); (3) *Stacking*: All possible combinations of the four individual classifiers were evaluated as base learners, while a Bayesian-optimized LR model was employed as the meta-learner and trained using the predictions generated by these base learners (44).

### 3.5 Bayesian hyperparameter optimization

Bayesian optimization (BO) with Gaussian process (GP) surrogates was employed to tune hyperparameters efficiently (45, 46). The objective function was defined as the classification accuracy on the validation set. For each classifier, the GP surrogate approximated the mapping from hyperparameters *x* to performance *f*(*x*), and an acquisition function guided the search by balancing exploration and exploitation:


$$\begin{array}{l} x_{i+1}=\arg\underset{x\in X}{\max}AF\left(x|f^{\prime }\right) \end{array}$$ (1)

A fixed budget of 32 optimization iterations was used per model. A fixed random seed ensured reproducibility. Detailed hyperparameter search spaces and the optimal settings obtained through Bayesian optimization for all evaluated classifiers are reported in the Supplementary Table S1.

### 3.6 Evaluation metrics

The performance of the ML models for seven-class skin lesion classification was evaluated using several commonly adopted metrics together with a normalized confusion matrix. These metrics provide a comprehensive assessment of the models' effectiveness, robustness, and discriminatory ability across different skin lesion categories. The confusion matrix represents a fundamental tool for analyzing classification outcomes by comparing the predicted labels with the corresponding ground-truth labels. In this study, normalization was applied to the confusion matrix to facilitate class-wise interpretation and provide clearer comparisons across lesion categories with different sample distributions. The evaluation metrics were derived from the standard confusion-matrix components, namely True Positives (TP), True Negatives (TN), False Positives (FP), and False Negatives (FN).

In skin cancer classification, the confusion matrix compares the predicted values, which represent the classified skin cancer types, with the actual values, which represent the true labels as follows:

- **TP**: Correct classification of a target class.
- **TN**: Correct classification of samples not belonging to the target class.
- **FP**: Non-target samples misclassified as the target class.
- **FN**: Target class samples misclassified as other classes.

These values form the basis for deriving key performance metrics, including accuracy, precision_macro_, recall_macro_, F1-score_macro_, and ROC-AUC_macro_. Due to class imbalance, macro-averaged metrics were adopted to assign equal importance to all classes. This ensures balanced performance evaluation across the seven lesion types. These metrics are mathematically defined as follows:


$$\begin{array}{l} \text{Accuracy}=\frac{TP+TN}{TP+TN+FP+FN} \end{array}$$ (2)

$$\begin{array}{l} {\text{Precision}}_{\text{macro}}=\frac{1}{7}\overset{7}{\underset{i=1}{\sum }}\frac{TP_{i}}{TP_{i}+FP_{i}} \end{array}$$ (3)

$$\begin{array}{l} {\text{Recall}}_{\text{macro}}=\frac{1}{7}\overset{7}{\underset{i=1}{\sum }}\frac{TP_{i}}{TP_{i}+FN_{i}} \end{array}$$ (4)

$$\begin{array}{l} {\text{F1-score}}_{\text{macro}}=\frac{1}{7}\overset{7}{\underset{i=1}{\sum }}\frac{2\times {\text{Precision}}_{i}\times {\text{Recall}}_{i}}{{\text{Precision}}_{i}+{\text{Recall}}_{i}} \end{array}$$ (5)

$$\begin{array}{l} {\text{ROC-AUC}}_{\text{macro}}=\frac{1}{7}\overset{7}{\underset{i=1}{\sum }}{\text{AUC}}_{i} \end{array}$$ (6)

where each class-wise area under the ROC curve is defined as


$$\begin{array}{l} {\text{AUC}}_{i}=\int_{0}^{1}{\text{TPR}}_{i}\left(\text{FPR}\right)d\left(\text{FPR}\right) \end{array}$$ (7)

and


$$\begin{array}{l} {\text{TPR}}_{i}=\frac{TP_{i}}{TP_{i}+FN_{i}},\;{\text{FPR}}_{i}=\frac{FP_{i}}{FP_{i}+TN_{i}} \end{array}$$ (8)

### 3.7 Visual interpretation via Grad-CAM

To enhance model transparency, Gradient-weighted Class Activation Mapping (Grad-CAM) (47) was employed to provide visual explanations for the predictions generated by the ViT model. Since the ViT architecture processes images as sequences of patch tokens rather than producing conventional convolutional feature maps, the Grad-CAM computation was adapted accordingly. Specifically, the output representations from the last transformer encoder layer were reshaped from the token sequence (excluding the classification token) back into a two-dimensional spatial grid corresponding to the original 3 × 3 patch layout. The gradients of the predicted class score with respect to these reshaped feature maps were computed via backpropagation. Global average pooling of these gradients produced channel-wise importance weights, which were used to generate a weighted combination of the feature maps. A ReLU activation was applied to retain only positive contributions, yielding a class-discriminative heatmap. The resulting Grad-CAM heatmap was then min-max normalized and resized to match the original input image resolution. Finally, the normalized heatmap was overlaid on the original skin lesion image using alpha blending to highlight the most influential regions contributing to the model's decision. It should be noted that Grad-CAM interprets the fine-tuned ViT feature extractor that supplies the fused representations to the downstream ensemble, since the stacked SVM+KNN ensemble operates on these features and does not itself produce spatial gradients. Accordingly, the predicted-class labels reported in the Grad-CAM visualizations correspond to the ViT softmax predictions rather than to the final ensemble output.

## 4 Results

The proposed ML-CAD framework for skin lesion classification was implemented using Python. The core libraries included Scikit-learn for preprocessing, ML models, and evaluation, and TensorFlow with vit_keras for developing and training the ViT model. All experiments were conducted on a computer with an Intel Core i7-12700H CPU (20 cores), 16 GB DDR4 RAM, and an NVIDIA RTX 4060 GPU with 8 GB VRAM. The experimental results in this work are divided into three main sections: fine-tuned ViT model, Bayesian-optimized individual ML models, and Bayesian-optimized EL models. All models evaluated in this study, including the fine-tuned ViT, individual ML, and EL models, were trained and evaluated using a unified stratified split of 70% training, 15% validation, and 15% testing. The validation and held-out test partitions consist exclusively of original, non-augmented, non-synthetic samples and therefore preserve the natural (imbalanced) HAM10000 class distribution; the per-partition class counts are reported in Table 1. Consequently, the reported metrics characterize performance under a clinically realistic class prevalence rather than an artificially balanced test distribution.

### 4.1 Fine-tuned ViT model

In the first experiment, the performance of the fine-tuned ViT model on multimodal skin lesion classification was evaluated. As summarized in Table 3, the model achieved accuracy, precision, recall, F1-score, and ROC-AUC values of 95.02%, 95.08%, 95.02%, 95.00%, and 99.66%, respectively. The corresponding training and validation curves are provided in the Supplementary Figure S1.

**Table 3:** Performance comparison of all evaluated models for seven-class skin lesion classification.

Model	Acc. (%)	Prec. (%)	Rec. (%)	F1 (%)	AUC (%)
Fine-tuned ViT	95.02	95.08	95.02	95.00	99.66
DT	95.43	95.43	95.43	95.43	97.39
LR	97.87	97.87	97.87	97.87	99.92
KNN	98.31	98.31	98.31	98.31	99.73
SVM	98.28	98.28	98.28	98.28	99.90
RF	97.93	97.93	97.93	97.92	99.89
ET	98.00	98.00	98.00	97.99	99.91
XGB	98.05	98.05	98.05	98.05	**99.93**
LGBM	98.11	98.11	98.11	98.11	**99.93**
SVM+KNN	**98.55**	**98.55**	**98.55**	**98.55**	99.88
SVM+KNN+DT	98.52	98.52	98.52	98.52	99.88
SVM+LR+KNN+DT	98.45	98.45	98.45	98.45	99.89

Only the top stacking combinations are shown; all variants achieved 98.01%–98.55% accuracy. Bold values indicate the best result obtained in each column.

### 4.2 Bayesian-optimized individual ML models

In the second experiment, the performance of the Bayesian-optimized individual ML models (SVM, DT, KNN, and LR) was evaluated using the multimodal feature representations extracted by the fine-tuned ViT model. The classification results are summarized in Table 3. Overall, all individual ML models demonstrated strong performance, confirming the effectiveness of the extracted multimodal features for skin lesion classification.

Among the evaluated models, KNN achieved the highest classification performance, with accuracy, precision, recall, and F1-score of 98.31%, along with a ROC-AUC of 99.73%. The SVM model delivered highly comparable results, achieving 98.28% across accuracy, precision, recall, and F1-score, with a ROC-AUC of 99.90%. LR ranked third, achieving 97.87% for accuracy, precision, recall, and F1-score, while attaining the highest ROC-AUC among the individual models at 99.92%. In contrast, DT achieved the lowest performance, with accuracy, precision, recall, and F1-score of 95.43%, and a ROC-AUC of 97.39%.

### 4.3 Ensemble learning models

In the third experiment, Bayesian-optimized EL models based on bagging, boosting, and stacking strategies were evaluated for multimodal skin lesion classification. The corresponding results are summarized in Table 3.

#### 4.3.1 Bagging models

In this section, the performance of two popular bagging models, Bayesian-optimized RF and ET, is evaluated. The results in Table 3 indicate strong similarity in performance between the two models, with the Bayesian-optimized ET model slightly outperforming the Bayesian-optimized RF model, achieving 98.00% accuracy, 98.00% precision, 98.00% recall, 97.99% F1-score, and 99.91% ROC-AUC, whereas the Bayesian-optimized RF model achieved 97.93% accuracy, 97.93% precision, 97.93% recall, 97.92% F1-score, and 99.89% ROC-AUC. The marginal performance difference between the two models can be attributed to their shared bagging mechanism, where both build ensembles of decision trees on bootstrapped subsets. However, ET introduces additional randomization through random split thresholds, which may provide slight gains in generalization on the multimodal feature space.

#### 4.3.2 Boosting models

In this section, the performance of two popular boosting models, Bayesian-optimized XGB and LGBM, is explored. The results in Table 3 show the Bayesian-optimized LGBM model slightly outperforming the Bayesian-optimized XGB model, achieving an accuracy of 98.11%, with precision, recall, and F1-score consistently reaching 98.11%, along with a ROC-AUC of 99.93%. The superior performance of LGBM can be attributed to its leaf-wise tree growth strategy and histogram-based algorithms, which enable more efficient learning on the high-dimensional fused feature representations. Both boosting models outperformed the bagging models, reflecting the sequential error-correction mechanism inherent to boosting strategies.

#### 4.3.3 Stacking models

To further improve and stabilize performance, this study combines various individual ML models using a stacking strategy. A set of ensembles was constructed encompassing all possible combinations of individual ML models (SVM, DT, KNN, and LR). Overall, the stacking method produced more robust and stable models, achieving high classification accuracies ranging from 98.55% to 98.01%, as shown in Table 3. Among all stacked models, the stacked SVM+KNN model achieved the highest overall performance, with 98.55% accuracy, 98.55% precision, 98.55% recall, 98.55% F1-score, and 99.88% ROC-AUC. It was followed by the stacked SVM+KNN+DT model, which demonstrated highly comparable performance, achieving 98.52% across accuracy, precision, recall, and F1-score, with a ROC-AUC of 99.88%. The stacked SVM+LR+KNN+DT model also demonstrated highly comparable performance, achieving 98.45% across accuracy, precision, recall, and F1-score, with a ROC-AUC of 99.89%.

These results highlight the effectiveness of the stacking strategy in leveraging the complementary strengths of multiple optimized learners. In particular, ensembles incorporating SVM consistently achieved superior or highly comparable performance, suggesting its pivotal role in defining optimal decision boundaries within the multimodal feature space. The integration of SVM with other models such as KNN, DT, and LR enhances both global boundary optimization and local decision refinement, leading to improved classification robustness and stability. In contrast, stacked models that excluded SVM generally exhibited slightly lower performance, further emphasizing its contribution to the overall ensemble effectiveness.

A comprehensive comparison of all ML models developed in this study demonstrates the consistent superiority of stacking models over individual, bagging, and boosting approaches, as summarized in Table 3. This performance gain can be attributed to the stacking strategy's ability to integrate the complementary strengths of multiple optimized learners, thereby enhancing decision diversity and reducing model variance. As a result, the stacked ensembles provide a more robust and stable predictive framework, particularly when handling the complex, nonlinear, and high-dimensional multimodal feature space.

**Figure 5:**
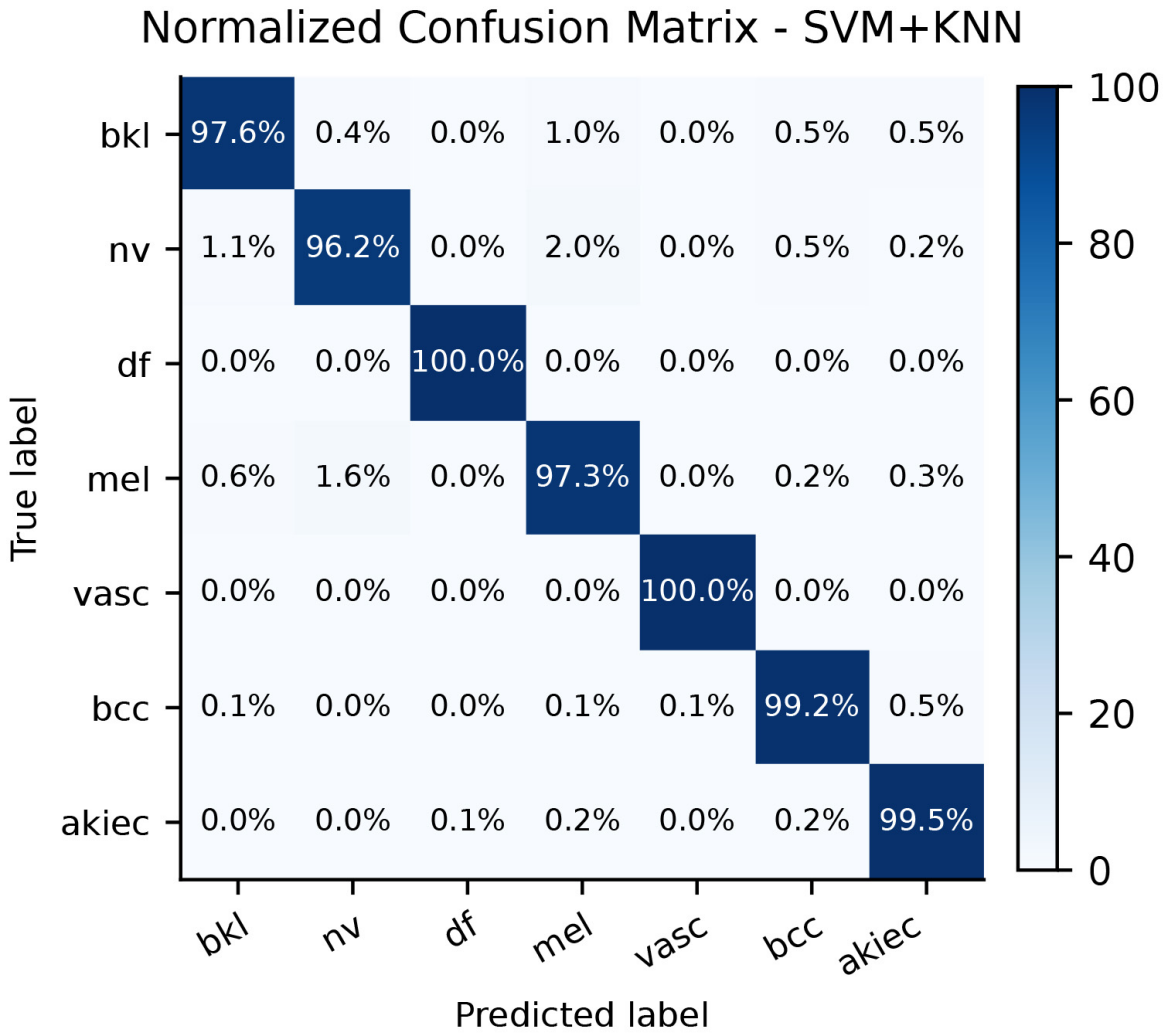
Normalized confusion matrix of the best-performing stacked SVM+KNN model for seven-class skin lesion classification.

In addition to the overall performance metrics, class-wise evaluation was conducted for the best-performing model (SVM+KNN) using sensitivity, specificity, precision, recall, F1-score, and ROC-AUC, as summarized in Table 4. Furthermore, the normalized confusion matrix of the SVM+KNN model is presented in Figure 5 to provide a detailed visualization of the classification performance across the seven skin lesion classes. The confusion matrices of the remaining top-performing models are provided in the Supplementary Figure S2.

**Table 4:** Class-wise performance of the best-performing stacked SVM+KNN model on the HAM10000 dataset.

Class	Sens. (%)	Spec. (%)	Prec. (%)	Rec. (%)	F1 (%)	AUC (%)
BKL	97.61	99.70	98.20	97.61	97.91	99.72
NV	96.22	99.67	97.98	96.22	97.09	99.71
DF	100.00	99.98	99.90	100.00	99.95	100.00
MEL	97.32	99.45	96.74	97.32	97.03	99.80
VASC	100.00	99.98	99.90	100.00	99.95	100.00
BCC	99.20	99.77	98.62	99.20	98.91	99.96
AKIEC	99.50	99.75	98.52	99.50	99.01	99.99

### 4.4 Ablation study

#### 4.4.1 Modality ablation

To systematically assess the contribution of each input modality, an extensive ablation study was conducted using different combinations of dermoscopic images and clinical metadata. The results are summarized in Table 5.

**Table 5:** Results of the ViT-based modality ablation study evaluating the individual and combined contributions of dermoscopic images and clinical metadata (age, Sex, and lesion localization) to skin lesion classification performance.

Input configuration	Acc. (%)	Prec. (%)	Rec. (%)	F1 (%)	AUC (%)
ViT classifier					
Metadata-only	40.59	39.64	40.59	35.82	78.68
Image-only	90.27	90.30	90.27	90.19	99.04
Images + age	91.11	91.22	91.11	91.05	99.23
Images + sex	92.05	92.04	92.04	91.99	99.23
Images + localization	93.54	93.48	93.54	93.49	99.52
Images + metadata	**95.02**	**95.08**	**95.02**	**95.00**	**99.66**
Best ensemble (stacked SVM+KNN)					
Metadata-only	49.42	48.53	49.42	47.93	83.58
Image-only	92.98	92.94	92.98	92.96	98.99
Images + age	93.18	93.20	93.18	93.18	99.25
Images + sex	93.67	93.65	93.67	93.65	99.17
Images + localization	94.43	94.42	94.43	94.42	99.31
Images + metadata	**98.55**	**98.55**	**98.55**	**98.55**	**99.88**

For each configuration, the performance of the best-performing stacked ensemble (SVM+KNN) trained on the corresponding fused features is also reported, to verify that the metadata benefit is preserved in the final modeling pipeline and not limited to the ViT classifier. Bold values indicate the best result in each column within each block (ViT classifier and best ensemble).

When the framework was trained exclusively on clinical metadata, it achieved an accuracy of 40.59%, indicating that demographic and clinical descriptors alone provide limited information for reliable skin lesion classification. In contrast, the image-only ViT model attained an accuracy of 90.27%, highlighting the important contribution of dermoscopic visual features to lesion characterization and classification.

The integration of individual metadata attributes with image features was associated with consistent improvements in classification performance relative to the image-only baseline, suggesting that clinical information provides complementary information to image features. Specifically, incorporating patient age increased the classification accuracy to 91.11%, while the inclusion of sex yielded an accuracy of 92.05%. Among the individual metadata variables, lesion localization was associated with the largest performance improvement, increasing the accuracy to 93.54%. This observation is clinically reasonable, as several skin lesion subtypes exhibit characteristic anatomical distributions, making lesion location a useful contextual factor for differential diagnosis.

The highest performance was obtained when all available metadata attributes were jointly integrated with dermoscopic image features. Under this multimodal configuration, the proposed framework attained an accuracy of 95.02%, a F1-score of 95.00%, and an ROC-AUC of 99.66%. This corresponds to an absolute accuracy increase of 4.75 percentage points compared with the image-only model. Overall, these findings suggest that clinical metadata provides complementary information beyond that contained in dermoscopic images alone. Furthermore, the progressive performance improvements observed across the ablation configurations indicate that the combined use of age, sex, and lesion localization can facilitate the learning of a more informative representation, contributing to improved skin lesion classification performance.

To confirm that the observed metadata benefit is retained in the final modeling pipeline rather than being specific to the ViT classification head, the best-performing stacked ensemble (SVM+KNN) was retrained and evaluated under each modality configuration using the corresponding fused features (lower block of Table 5). The ensemble consistently outperformed the ViT classifier across all configurations while reproducing the same monotonic trend: accuracy increased from 92.98% for the image-only setting to 98.55% when all metadata attributes were incorporated, an absolute improvement of 5.57 percentage points that mirrors the gain observed for the ViT classifier. Lesion localization again provided the largest single-attribute improvement among the individual metadata variables. These results confirm that the complementary value of clinical metadata is preserved in the final ensemble model and is not an artifact of the ViT classification head.

#### 4.4.2 Input resolution ablation

The results of the input resolution analysis are presented in Table 6. Classification performance improved consistently as the input resolution increased from 48 × 48 to 128 × 128 and 224 × 224, with accuracy increasing from 95.02% to 95.57% and 95.78%, respectively. Similar trends were observed for the F1-score and ROC-AUC, indicating that higher-resolution images preserve additional diagnostically relevant information.

**Table 6:** Impact of input image resolution on the classification performance and computational cost of the ViT model.

Resolution	Acc. (%)	F1 (%)	AUC (%)	Train time	Params / FLOPs
48 × 48	95.02	95.00	99.66	**45 min**	**85.76M/0.17G**
128 × 128	95.57	95.55	99.77	2.5 h	85.76M/1.22G
224 × 224	**95.78**	**95.78**	**99.78**	6.5 h	85.76M/4.55G

Bold values indicate the best result in each column: the highest accuracy, F1-score, and ROC-AUC (224 × 224 resolution) and the lowest computational cost in training time and FLOPs (48 × 48 resolution).

However, these gains were accompanied by substantially higher computational costs. Training time increased from 45 minutes at 48 × 48 to 2.5 hours and 6.5 hours at 128 × 128 and 224 × 224, respectively, while computational complexity increased from 0.17G to 1.22G and 4.55G FLOPs. Notably, the overall accuracy gain from 48 × 48 to 224 × 224 was only 0.76 percentage points, indicating a clear trade-off between predictive performance and computational efficiency. Despite its lower resolution, the 48 × 48 configuration remained highly competitive, achieving 95.02% accuracy and 99.66% ROC-AUC while requiring substantially fewer computational resources. Therefore, although 224 × 224 achieved the best performance, the 48 × 48 resolution was adopted in this study as it provides a favorable balance between classification performance and computational efficiency.

### 4.5 Grad-CAM analysis

**Figure 6:**
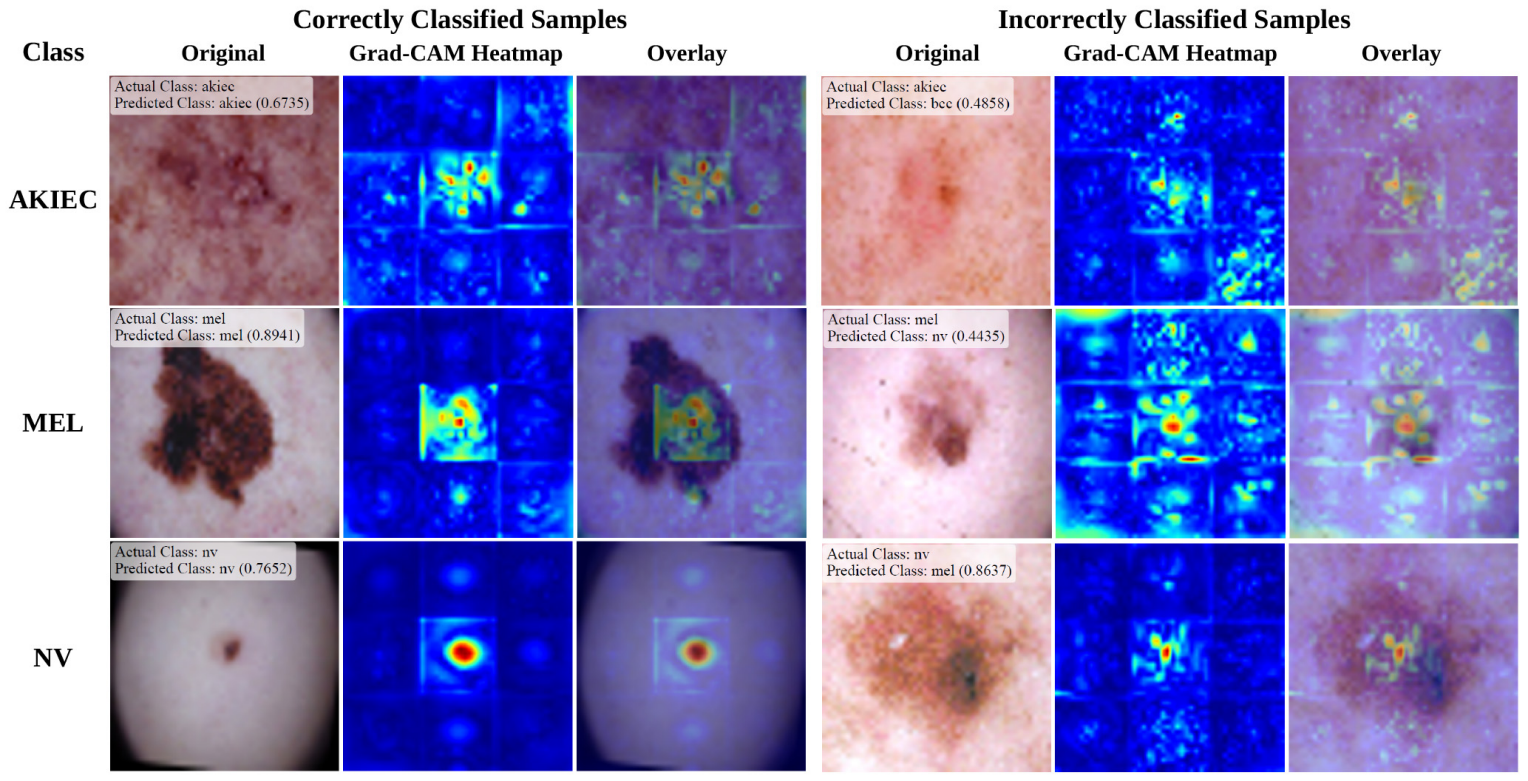
Representative Grad-CAM visualizations for MEL (malignant), AKIEC (precancerous), and NV (benign) lesions. Correctly classified and misclassified examples are shown with the original image, Grad-CAM heatmap, and overlay, highlighting the regions that contributed most strongly to the model's predictions.

To improve the interpretability of the proposed ML-CAD framework, Grad-CAM was employed to visualize the image regions that contributed most strongly to the model's predictions. Figure 6 presents representative examples from three clinically important lesion categories selected to reflect the major diagnostic groups encountered in dermoscopic practice: MEL as a representative malignant lesion, AKIEC as a representative precancerous lesion, and NV as a representative benign lesion. For each category, one correctly classified and one misclassified example are presented. Here, the correctly classified and misclassified labels refer to the predictions of the ViT feature extractor on which Grad-CAM is computed, rather than to the final SVM+KNN ensemble, as the ensemble does not produce spatial gradient maps. The original dermoscopic image, the corresponding Grad-CAM heatmap, and the overlay visualization are shown to facilitate comparison of the model's attention patterns and to provide insight into the factors influencing both successful and erroneous predictions.

As illustrated in Figure 6, correctly classified samples exhibit a strong concentration of attention within the lesion boundaries. In the AKIEC example, the activation map focuses on the atypical erythematous region, while in the MEL case, the highlighted areas correspond to the pigmented and structurally irregular portions of the lesion. Similarly, for NV, the model concentrates on the central nevus region with minimal activation in the surrounding healthy skin. These observations indicate that the proposed framework relies primarily on clinically relevant lesion characteristics rather than background artifacts.

In contrast, the misclassified samples reveal a different attention pattern. Although the model generally remains focused on the lesion area, the highlighted regions appear less discriminative and fail to capture some of the morphological characteristics required for accurate differentiation. For example, the misclassified MEL and NV cases demonstrate substantial overlap in the activated pigmented regions, reflecting the well-known visual similarity between melanocytic lesion categories. Likewise, the misclassified AKIEC example shows attention concentrated on local lesion structures that may share appearance characteristics with other classes, leading to an incorrect prediction.

Overall, the Grad-CAM visualizations suggest that model errors are primarily associated with inter-class visual similarity rather than attention to irrelevant image regions. The consistent localization of diagnostically meaningful lesion structures in both correctly classified and misclassified examples supports the interpretability of the proposed framework and provides additional evidence that the model's decisions are driven by clinically relevant image features. Additional examples from the remaining lesion categories demonstrate similar attention patterns (see Supplementary Figures S3, S4).

## 5 Discussion

This study introduces a multimodal ML-CAD framework that integrates dermoscopic images with clinical metadata to address persistent challenges in seven-class skin lesion classification. By aligning AI techniques with the principles of multimodal medical data fusion, the framework is intended as a research contribution toward interpretable decision support; translation into routine dermatological practice would require the external and prospective validation outlined in Section 5.5. Table 7 provides a structured comparison between the proposed framework and recent studies on seven-class HAM10000 classification under balanced settings. The comparison encompasses CNN-based, transformer-based, EL, and hybrid models, highlighting variations in imbalance handling techniques, feature extraction strategies, classifiers, and explainability support. As these studies differ in data partitioning, balancing, and evaluation protocol, this comparison is indicative rather than a controlled head-to-head benchmark.

**Table 7:** Comparison with recent methods on seven-class HAM10000 classification.

Reference	Split strategy	Modality	Balancing	Features	Classifier	Acc.	XAI
Shetty et al. (14)	Hold-out	Dermoscopy images	Data augmentation	CNN	Softmax	95.18%	None
Alwakid et al. (17)	Hold-out	Dermoscopy Images	Data augmentation	ESRGAN + ResNet - 50	Softmax	86.00%	None
Ali et al. (15)	Hold-out	Dermoscopy images	Data augmentation	EfficientNet - B4	Softmax	87.90%	None
Abbas and Elbehiery (18)	Hold-out	Dermoscopy images	Data augmentation	Xception	Softmax	91.48%	None
Velaga et al. (12)	Hold-out	Dermoscopy Images	SMOTE	Hand-crafted	RF	95.60%	None
Asaduzzaman et al. (19)	Hold-out	Dermoscopy images	GAN + SMOTE	CNN	Voting ensemble	94.10%	None
Khan et al. (21)	Hold-out	Dermoscopy images	Data augmentation	InceptionV3 + ResNet50	Voting ensemble	94.17%	None
Natha et al. (22)	Hold-out	Dermoscopy images	Data augmentation	Hand-crafted	Max voting (RF + SVM + MLPN)	94.70%	None
Yang et al. (7)	Hold-out	Dermoscopy images	Data augmentation	Vision transformer	Softmax	94.10%	Attention maps
Aladhadh et al. (23)	Hold-out	Dermoscopy images	Data augmentation	Medical vision transformer	MLP	96.14%	Grad-CAM
Khan et al. (20)	Hold-out	Dermoscopy images	Data augmentation	DenseNet121 + CNN	Softmax	93.24%	None
Fiaz et al. (13)	Hold-out	Dermoscopy images	Data augmentation	U-Net + EfficientNet-B0	Softmax	85.57%	Grad-CAM
Tran-Van and Le (29)	Hold-out	Dermoscopy images + metadata	None	DenseNet121 + Cross-attention fusion	Softmax	95.73%	None
Saeed et al. (28)	Hold-out	Dermoscopy images + Metadata	SMOTE	ResNet50 + Xception + EfficientNetB0	Adaptive weighted ensemble	93.20%	Grad-CAM
Govindu et al. (16)	Hold-out	Dermoscopy images	SMOTE	Hybrid CNN	Softmax	96.40%	None
This study	Hold-out	Dermoscopy images + metadata	Multimodal	Fine-tuned ViT	Stacked	98.55%	Grad-CAM

The listed studies differ in data partitioning, balancing strategy, and evaluation protocol; the comparison is therefore indicative rather than a controlled head-to-head benchmark.

### 5.1 Class imbalance handling

Class imbalance remains a central challenge in seven-class skin lesion classification using HAM10000. Most prior studies address this issue through conventional image-level augmentation (7, 14, 15, 17, 18, 20, 21, 23), which increases data volume but does not introduce genuine semantic diversity. A limited number of works adopt SMOTE-based oversampling (12, 16), which is more suitable for structured data than high-dimensional image representations. Other approaches employ GAN-based generation (19), offering realistic samples at the cost of increased computational complexity and training instability. In contrast, the proposed study adopts a structured multimodal balancing strategy that jointly addresses skin lesion images and clinical metadata. Through class-wise SMOTENC, controlled augmentation, and cross-modal alignment, the framework achieves balanced classes while preserving clinical consistency, contributing to improved performance.

### 5.2 Feature extraction and representation

Feature extraction is a critical stage in skin lesion analysis, as classification performance depends heavily on the quality of learned representations. Early studies relied on hand-crafted features (12), which were limited in capturing complex lesion patterns. Later, CNN-based automatic feature extraction (14) and transfer learning with pretrained models (15, 18) improved representation learning. However, the performance of these models remained moderate compared to more advanced transformer (23) or hybrid architectures (16). Despite these advances, most prior methods remained image-centric and overlooked clinical metadata during feature extraction. In contrast, the proposed framework utilizes a fine-tuned ViT within a multimodal late-fusion strategy, enabling enriched cross-modal feature representations while maintaining architectural efficiency.

### 5.3 Classifier design and optimization

In the classification stage, most prior studies relied primarily on the fully connected layer for final classification (7, 14, 16–18, 20). A smaller number of works employed ML classifiers (12, 23). However, hyperparameter tuning in these studies was often manual or limited, which may restrict optimal performance. In contrast, the proposed study applies Bayesian optimization and ensemble aggregation to enhance robustness and predictive stability beyond conventional end-to-end DL classification schemes.

### 5.4 Interpretability and clinical relevance

Model interpretability is essential in clinical skin lesion analysis to ensure transparency and support physician trust. However, most prior studies primarily focused on improving classification accuracy through increasingly complex architectures without sufficient attention to explaining model decisions (14–22). As summarized in Table 7, the majority of comparable HAM10000 studies provide no interpretability or explainability analysis whatsoever. Only limited works provided visual interpretation using attention maps (7) or Grad-CAM (13, 23, 28). In contrast, the proposed study improves transparency by employing Grad-CAM to provide visual interpretability, systematically analyzing both correctly and incorrectly predicted samples across all seven lesion categories, providing deeper insight into model decision behavior. We emphasize that this constitutes the visual interpretability of predictions rather than a comprehensive, multi-method explainability framework. In the future, we plan to expand explainability analyses to include methods beyond Grad-CAM, such as attention rollout (Section 6). Even so, providing systematic Grad-CAM analysis across all seven classes already exceeds the interpretability reporting of most prior approaches, which is increasingly expected for trustworthy AI-driven diagnostic tools.

### 5.5 Limitations

Despite these strengths, certain limitations remain. The most important limitation, and the primary direction of our future work, is the absence of external and prospective validation. All results were obtained on a single, retrospectively collected dataset (HAM10000), and the framework has not been evaluated on independent cohorts or within a clinical workflow. Consequently, the reported metrics characterize internal held-out (single test-set) performance only and should not be interpreted as evidence of clinical readiness or generalizability to other populations, devices, or acquisition conditions. External validation on independent datasets such as ISIC 2019, Derm7pt, and prospective evaluation in real dermatological workflows are required before any claim of translational or clinical utility can be made. Additionally, the reduced 48 × 48 resolution, while computationally efficient, may discard fine-grained spatial features relevant to certain lesion types. Per-class performance analysis reveals that most residual errors occur among the visually similar melanocytic/keratinocytic classes (notably NV, MEL, and BKL), consistent with the MEL–NV confusion observed in the Grad-CAM analysis. Another limitation is the limited set of available clinical variables. The HAM10000 provides only patient age, sex, and lesion localization. Richer clinical context, such as lesion duration, evolution, symptoms (e.g., itching or bleeding), prior biopsy, and family history of skin cancer, is unavailable. Such variables carry diagnostic value, and incorporating them in future multimodal datasets could further improve discrimination, particularly among visually similar lesion classes. Relatedly, because only class-level metadata is available, the pairing of SMOTENC-generated metadata with same-class images is an approximation of true patient-level co-occurrence rather than a reconstruction of authentic patient records, which may introduce class-conditional feature associations not present in real clinical data. A further limitation concerns interpretability: the framework provides visual interpretability through Grad-CAM rather than a comprehensive, multi-method explainability framework, and extending this analysis remains future work.

## 6 Conclusion

This study proposed a multimodal ML-CAD framework for seven-class skin lesion classification, integrating dermoscopic images with clinical metadata through a structured five-phase pipeline. The framework addresses key limitations of existing approaches: class imbalance via multimodal SMOTENC-based balancing with cross-modal alignment, feature representation via ViT-based extraction with metadata fusion, and classification robustness via Bayesian-optimized stacked ensembles. On the HAM10000 dataset, the stacked SVM+KNN ensemble achieved 98.55% accuracy and 99.88% ROC-AUC, achieving competitive accuracy among recently reported methods under broadly similar balanced settings. Grad-CAM analysis confirmed that the model attends to clinically relevant lesion regions, supporting its potential for clinical decision support. By demonstrating how AI-driven multimodal fusion can deliver both improved performance and visual interpretability via Grad-CAM, the present work contributes to the broader goal of developing interpretable multimodal AI for dermatology. We reiterate, however, that the present evidence is based on a single retrospective dataset and that external and prospective clinical validation remain necessary before translation into real clinical practice.

The primary direction of future work is external and prospective validation: we will first evaluate the framework on independent datasets such as ISIC 2019 and Derm7pt to assess generalizability, and subsequently pursue prospective evaluation within real dermatological workflows, as these steps are prerequisites for any claim of clinical utility. Future work will also extend this framework by exploring advanced transformer architectures, such as the Data-Efficient Image Transformer and the Swin Transformer. Additionally, it will investigate more sophisticated augmentation strategies, including self-supervised approaches. We will also explore additional interpretability methods beyond Grad-CAM, such as attention rollout, to establish a more comprehensive explainability framework.

## Data Availability

The original contributions presented in the study are included in the article/supplementary material, further inquiries can be directed to the corresponding author/s.

## Footnotes

1 World Health Organization, “Cancer,” Available: https://www.who.int/news-room/fact-sheets/detail/cancer, Accessed: May 1, 2026.

## References

[1] Ajabani D Shaikh ZA Yousef A Ali K Albahar MA. Enhancing skin lesion classification: a CNN approach with human baseline comparison. PeerJ Comput Sci. (2025) 11:e2795. doi: 10.7717/peerj-cs.2795 40567717 PMC12192895

[2] Ray A Sarkar S Schwenker F Sarkar R. Decoding skin cancer classification: perspectives, insights, and advances through researchers' lens. Sci Rep. (2024) 12:14. doi: 10.1038/s41598-024-81961-3 39695157 PMC11655883

[3] Brochez L Volkmer B Hoorens I Garbe C Röcken M Schz J . Skin cancer in Europe today and challenges for tomorrow. J Eur Acad Dermatol Venereol. (2025) 2:39. doi: 10.1111/jdv.20368 39377431

[4] Dildar M Akram S Irfan M Khan HU Ramzan M Mahmood AR . Skin cancer detection: A review using deep learning techniques. Int J Environ Res Public Health. (2021) 18:5479. doi: 10.3390/ijerph18105479 34065430 PMC8160886

[5] Rajarajeswari S Prassanna J Quadir MA Jackson JC Sharma S Rajesh B. Skin cancer detection using deep learning. Res J Pharm Technol. (2022) 15:4519–25. doi: 10.52711/0974-360X.2022.00758

[6] Li H Pan Y Zhao J Zhang L. Skin disease diagnosis with deep learning: a review. Neurocomputing. (2021) 464:364–93. doi: 10.1016/j.neucom.2021.08.096

[7] Yang G Luo S Greer P. A novel vision transformer model for skin cancer classification. Neural Proc Lett. (2023) 55:9335–51. doi: 10.1007/s11063-023-11204-5

[8] Himel GMS Islam MM Al-Aff KA Karim SI Sikder MKU. Skin cancer segmentation and classification using vision transformer for automatic analysis in dermatoscopy-based noninvasive digital system. Int J Biomed Imag. (2024) 2024:3022192. doi: 10.1155/2024/3022192 38344227 PMC10858797

[9] Bechelli S Delhommelle J. Machine learning and deep learning algorithms for skin cancer classification from dermoscopic images. Bioengineering. (2022) 9:97. doi: 10.3390/bioengineering9030097 35324786 PMC8945332

[10] Khan MA Ali MD Mazhar T Shahzad T Rehman WU Shahid M . An advanced deep learning framework for skin cancer classification. Rev Socionetw Strat. (2025) 19:111–30. doi: 10.1007/s12626-025-00181-x

[11] Tahir M Naeem A Malik H Tanveer J Naqvi RA Lee SW. DSCC_Net: multi-classification deep learning models for diagnosing of skin cancer using dermoscopic images. Cancers. (2023) 15:2179. doi: 10.3390/cancers15072179 37046840 PMC10093058

[12] Velaga NK Vardineni VR Tupakula P Pamidimukkala JS. Skin cancer detection using the HAM10000 dataset: a comparative study of machine learning models. In: 2023 global conference on information technologies and communications (GCITC). IEEE (2023). p. 1–6. doi: 10.1109/GCITC60406.2023.10425889

[13] Fiaz M Shoaib Khan MB Khan AH Bilal A Abdullah M Darem AA . An explainable hybrid deep learning framework for precise skin lesion segmentation and multi-class classification. Front Med. (2025) 12:1681542. doi: 10.3389/fmed.2025.1724427 41158448 PMC12554749

[14] Shetty B Fernandes R Rodrigues AP Chengoden R Bhattacharya S Lakshmanna K. Skin lesion classification of dermoscopic images using machine learning and convolutional neural network. Sci Rep. (2022) 12:18134. doi: 10.1038/s41598-022-22644-9 36307467 PMC9616944

[15] Ali MS Miah MS Haque J Rahman MM Islam MK. An enhanced technique of skin cancer classification using deep convolutional neural network with transfer learning models. Mach Learn Applic. (2021) 5:100036. doi: 10.1016/j.mlwa.2021.100036

[16] Govindu S Devi OR Sitharam M Koreddi V Kumar MK Sunitha M. Cutting-edge CNN-based skin cancer detection with batch normalization and advanced imbalance learning for superior medical image classification. Biomed Signal Process Control. (2026) 113:108929. doi: 10.1016/j.bspc.2025.108929

[17] Alwakid G Gouda W Humayun M Sama NU. Melanoma detection using deep learning-based classifications. Healthcare. (2022) 10:2481. doi: 10.3390/healthcare10122481 36554004 PMC9777935

[18] Abbas A Elbehiery H. A robust skin cancer classification using deep learning. Sustain Mach Intell J. (2025) 10:3. doi: 10.61356/SMIJ.2025.10507

[19] Asaduzzaman A Thompson CC Uddin MJ. Machine learning approaches for skin neoplasm diagnosis. ACS Omega. (2024) 9:32853–63. doi: 10.1021/acsomega.4c03640 39100361 PMC11292652

[20] Khan AR Mujahid M Alamri FS Saba T Ayesha N. Early-stage melanoma cancer diagnosis framework for imbalanced data from dermoscopic images. Microsc Res Tech. (2025) 88:797–809. doi: 10.1002/jemt.24736 39573895

[21] Khan MA Alam S Ahmed W. Enhanced skin cancer diagnosis via deep convolutional neural networks with ensemble learning. SN Comput Sci. (2025) 6:124. doi: 10.1007/s42979-024-03581-y

[22] Natha P Tera SP Chinthaginjala R Rab SO Narasimhulu CV Kim TH. Boosting skin cancer diagnosis accuracy with ensemble approach. Sci Rep. (2025) 15:1290. doi: 10.1038/s41598-024-84864-5 39779772 PMC11711234

[23] Aladhadh S Alsanea M Aloraini M Khan T Habib S Islam M. An effective skin cancer classification mechanism via medical vision transformer. Sensors. (2022) 22:4008. doi: 10.3390/s22114008 35684627 PMC9182815

[24] Xin C Liu Z Zhao K Miao L Ma Y Zhu X . An improved transformer network for skin cancer classification. Comput Biol Med. (2022) 149:105939. doi: 10.1016/j.compbiomed.2022.105939 36037629

[25] Rao MK Krishna GS Supriya K Sorgile M. LesionAid: vision transformers-based skin lesion generation and classification-a practical review. Multimed Tools Appl. (2025) 84:41405–26. doi: 10.1007/s11042-025-20797-z

[26] Tang P Yan X Nan Y Xiang S Krammer S Lasser T. FusionM4Net: a multi-stage multi-modal learning algorithm for multi-label skin lesion classification. Med Image Anal. (2022) 76:102307. doi: 10.1016/j.media.2021.102307 34861602

[27] Khurshid M Singh R Vatsa M. Multimodal dual-stage feature refinement for robust skin lesion classification. Sci Rep. (2025) 15:37775. doi: 10.1038/s41598-025-14839-7 41162437 PMC12572157

[28] Saeed MA Afify YM Badr NL Helal NA. Multimodal deep learning ensemble framework for skin cancer detection. Sci Rep. (2025) 15:45660. doi: 10.1038/s41598-025-30534-z 41469782 PMC12753777

[29] Tran-Van NY Le KH A. multimodal skin lesion classification through cross-attention fusion and collaborative edge computing. Computer Med Imag Graph. (2025) 124:102588. doi: 10.1016/j.compmedimag.2025.102588 40561935

[30] Tschandl P Rosendahl C Kittler H. The HAM10000 dataset, a large collection of multi-source dermatoscopic images of common pigmented skin lesions. Sci Data. (2018) 5:1–9. doi: 10.1038/sdata.2018.161 30106392 PMC6091241

[31] Chawla NV Bowyer KW Hall LO Kegelmeyer WP. SMOTE synthetic minority over-sampling technique. J Artif Intell Res. (2002) 16:321–57. doi: 10.1613/jair.953

[32] Dewi R Sri Hayati R Saleh A Tanjung DYH Jinan A. Enhancing machine learning algorithm performance for PCOS diagnosis using SMOTENC on imbalanced data. JITK. (2025) 11:55–63. doi: 10.33480/jitk.v11i1.6676

[33] Buslaev A Iglovikov VI Khvedchenya E Parinov A Druzhinin M Kalinin AA. Albumentations: fast and flexible image augmentations. Information. (2020) 11:125. doi: 10.3390/info11020125

[34] Dosovitskiy A Beyer L Kolesnikov A Weissenborn D Zhai X Unterthiner T . An image is worth 16x16 words: transformers for image recognition at scale. arXiv preprint arXiv:2010.11929. (2020).

[35] Cortes C Vapnik V. Support-vector networks. Mach Learn. (1995) 20:273–97. doi: 10.1007/BF00994018

[36] Breiman L Friedman JH Olshen RA Stone CJ. Classification and Regression Trees. Belmont, CA, USA: Wadsworth International Group. (1984).

[37] Cover TM Hart PE. Nearest neighbor pattern classification. IEEE Trans Inf Theory. (1967) 13:21–7. doi: 10.1109/TIT.1967.1053964

[38] Cox DR. The regression analysis of binary sequences. J R Stat Soc Series B. (1958) 20:215–42. doi: 10.1111/j.2517-6161.1958.tb00292.x

[39] Saihood Q Sonu E. A practical framework for early detection of diabetes using ensemble machine learning models. Turkish J Electr Eng Comput Sci. (2023) 31:722–38. doi: 10.55730/1300-0632.4013

[40] Breiman L. Random forests. Mach Learn. (2001) 45:5–32. doi: 10.1023/A:1010933404324

[41] Geurts P Ernst D Wehenkel L. Extremely randomized trees. Mach Learn. (2006) 63:3–42. doi: 10.1007/s10994-006-6226-1

[42] Chen T Guestrin C. Xgboost: a scalable tree boosting system. In: Proceedings of the 22nd ACM SIGKDD international conference on knowledge discovery and data mining (2016). p. 785–794. doi: 10.1145/2939672.2939785

[43] Ke G Meng Q Finley T Wang T Chen W Ma W . LightGBM: a highly efficient gradient boosting decision tree. In: Advances in neural information processing systems. (2017). p. 3146–3154.

[44] Wolpert DH. Stacked generalization. Neural Netw. (1992) 5:241–59. doi: 10.1016/S0893-6080(05)80023-1

[45] Victoria AH Maragatham G. Automatic tuning of hyperparameters using Bayesian optimization. Evolv Syst. (2021) 12:217–23. doi: 10.1007/s12530-020-09345-2

[46] Ilemobayo JA Durodola O Alade O Awotunde OJ Olanrewaju AT Falana O . Hyperparameter tuning in machine learning: a comprehensive review. J Eng Res Rep. (2024) 26:388–95. doi: 10.9734/jerr/2024/v26i61188

[47] Selvaraju RR Cogswell M Das A Vedantam R Parikh D Batra D. Grad-CAM: visual explanations from deep networks via gradient-based localization. In: Proceedings of the IEEE international conference on computer vision (ICCV) (2017). p. 618–626. doi: 10.1109/ICCV.2017.74

