# Supplementary material for: Interpretable multimodal fusion for skin lesion classification using dermoscopic images and patient metadata
Source: Front Med (Lausanne). 2026 Jul 29;13:1871689. doi: 10.3389/fmed.2026.1871689 (PMC13461512; doi:10.3389/fmed.2026.1871689)
Supplement: Supplementary file 1 [file Supplementary_file_1.pdf]

Supplementary Material

1 SUPPLEMENTARY TABLES AND FIGURES

This Supplementary Material contains the supporting tables and figures referenced in the main manuscript. Supplementary Table S1 reports the full Bayesian optimization search spaces and the selected hyperparameters for all evaluated classifiers. Supplementary Figure S1 shows the training and validation convergence of the fine-tuned ViT feature extractor. Supplementary Figure S2 presents the normalized confusion matrices of the remaining top-performing stacking ensembles. Supplementary Figures S3 and S4 provide additional Grad-CAM visual explanations for correctly classified and misclassified samples, respectively.

Table S1. Bayesian optimization search spaces and selected optimal hyperparameters for all evaluated machine learning classifiers.

Model	Hyperparameter	Search Range	Optimal Value
SVM	C	$[10^{-3}, 10^{3}]$ (log)	8.542
	kernel	{linear, poly, rbf, sigmoid}	rbf
	degree	[2,5]	5
	gamma	$[10^{-4}, 1]$	0.017
DT	criterion	{gini, entropy}	entropy
	splitter	{best, random}	random
	max_depth	[5,20]	18
	min_samples_split	[2,10]	2
KNN	n_neighbors	[1,29]	28
	weights	{uniform, distance}	distance
	metric	{minkowski, euclidean, manhattan}	manhattan
LR	C	$[10^{-3}, 10^{3}]$ (log)	0.255
	penalty	{l1,l2,elasticnet}	l2
	max_iter	[100,2000]	2000
RF	n_estimators	[50,300]	300
	criterion	{gini, entropy}	gini
	max_depth	[5,20]	19
	min_samples_split	[2,10]	2
ET	n_estimators	[50,300]	158
	criterion	{gini, entropy}	gini
	max_depth	[3,20]	19
	min_samples_split	[2,10]	3
XGB	n_estimators	[50,300]	300
	sampling_method	{uniform, gradient-based}	uniform
	max_depth	[5,20]	5
	learning_rate	$[0.01, 0.3]$ (log)	0.186
	eval_metric	{mlogloss,error,auc}	auc
LGBM	n_estimators	[50,300]	199
	objective	{multiclass,multiclassova}	multiclassova
	max_depth	[5,20]	20
	learning_rate	$[0.01, 0.3]$ (log)	0.3
	num_leaves	[20,150]	20

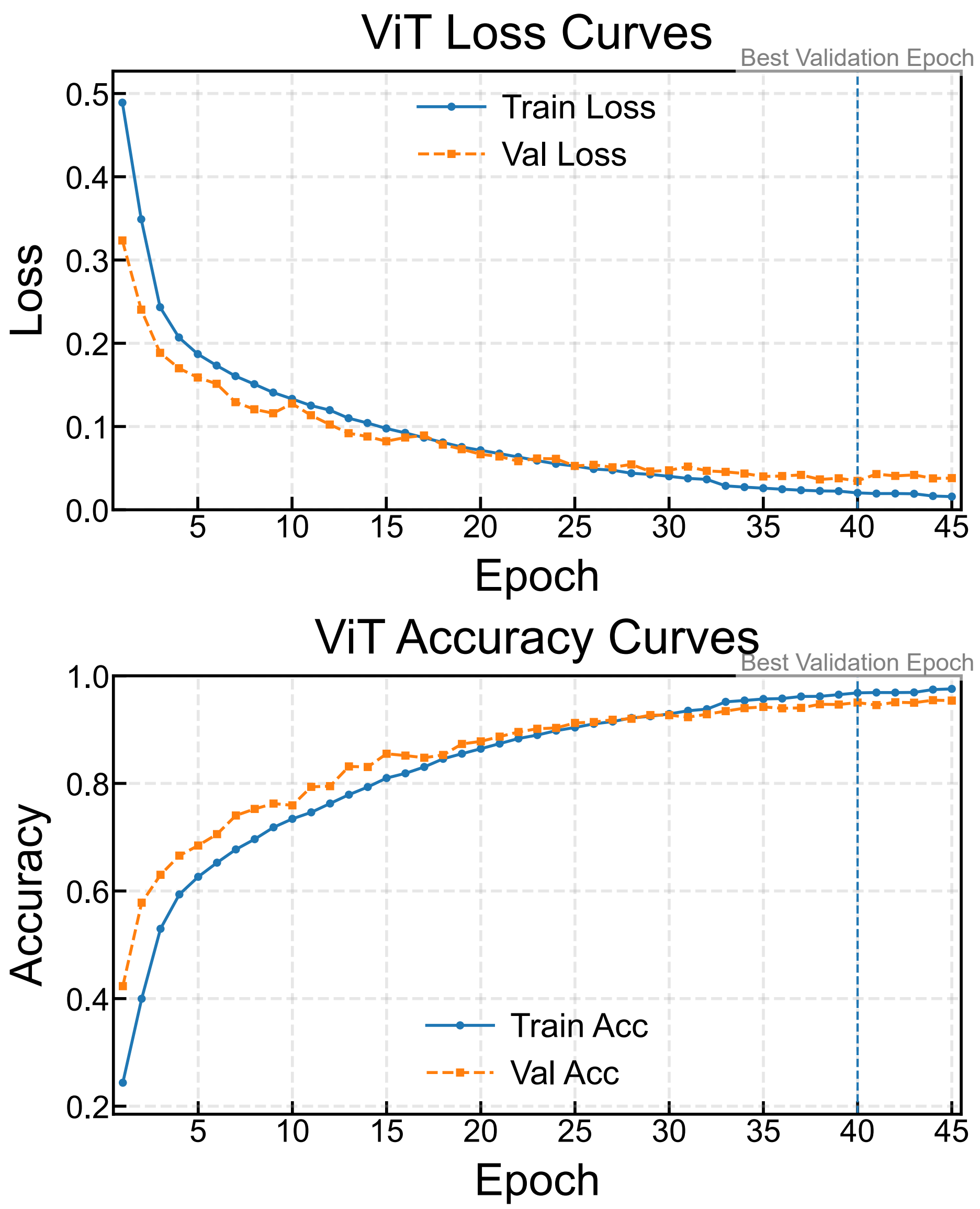

Figure S1. Training and validation performance of the fine-tuned ViT model in terms of loss and accuracy.

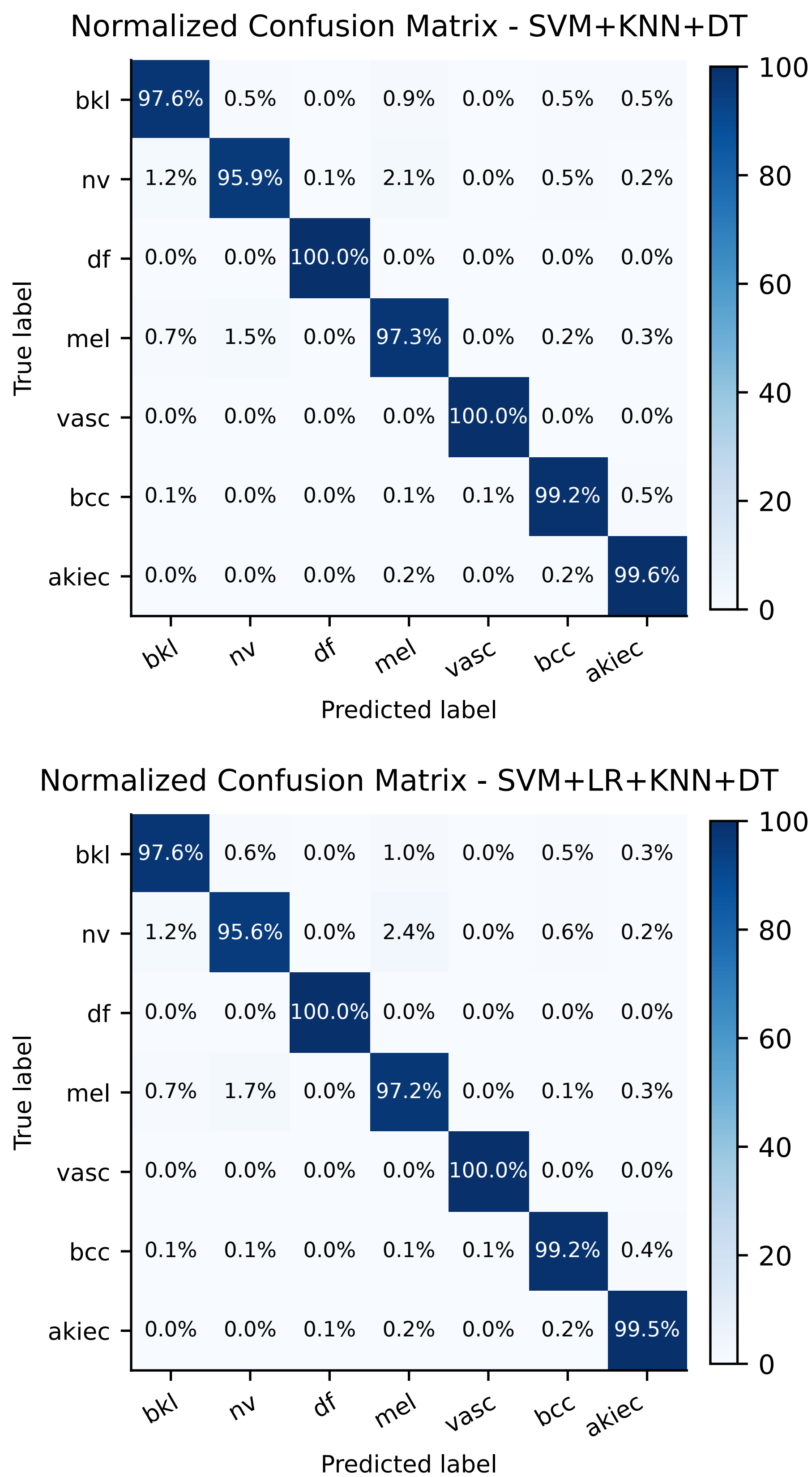

Figure S2. Normalized confusion matrices of the second- and third-best stacking ensembles evaluated on the held-out test set. The upper panel corresponds to the SVM+KNN+DT ensemble, whereas the lower panel corresponds to the SVM+LR+KNN+DT ensemble.

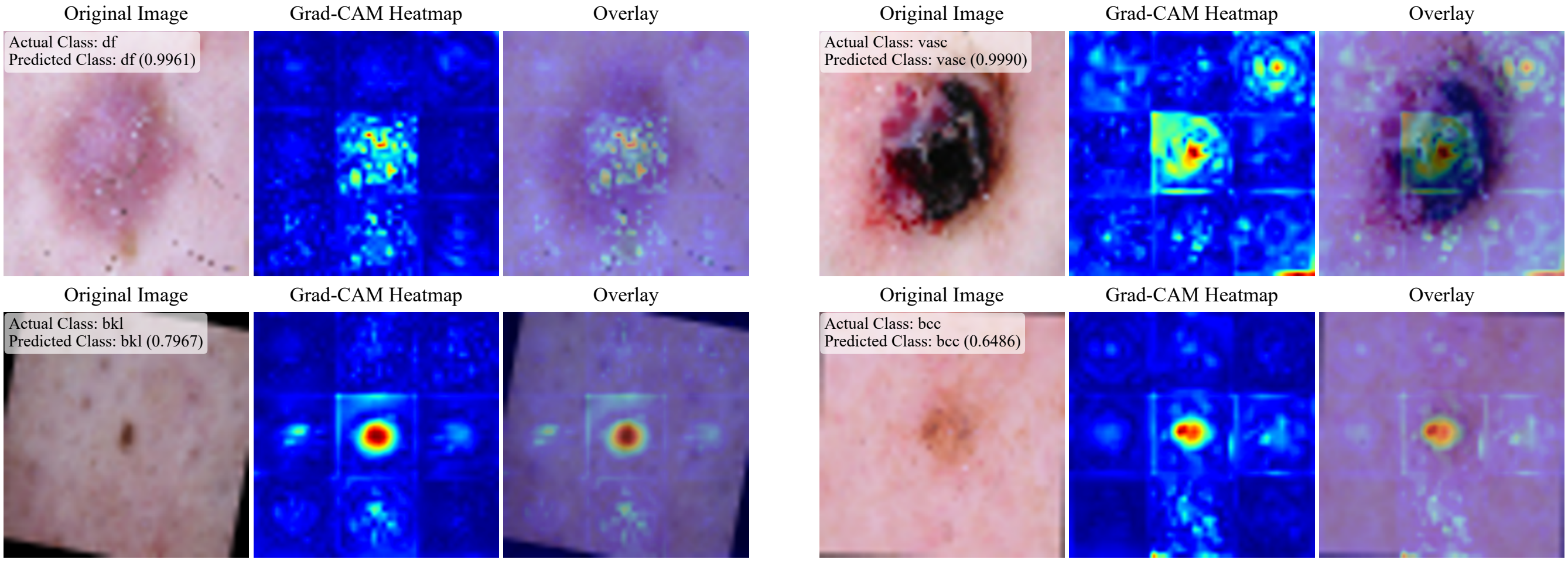

Figure S3. Additional Grad-CAM visual explanations for correctly classified samples not shown in the main manuscript. From left to right and top to bottom: DF, VASC, BKL, and BCC. Each example includes the original image, Grad-CAM heatmap, and overlay visualization.

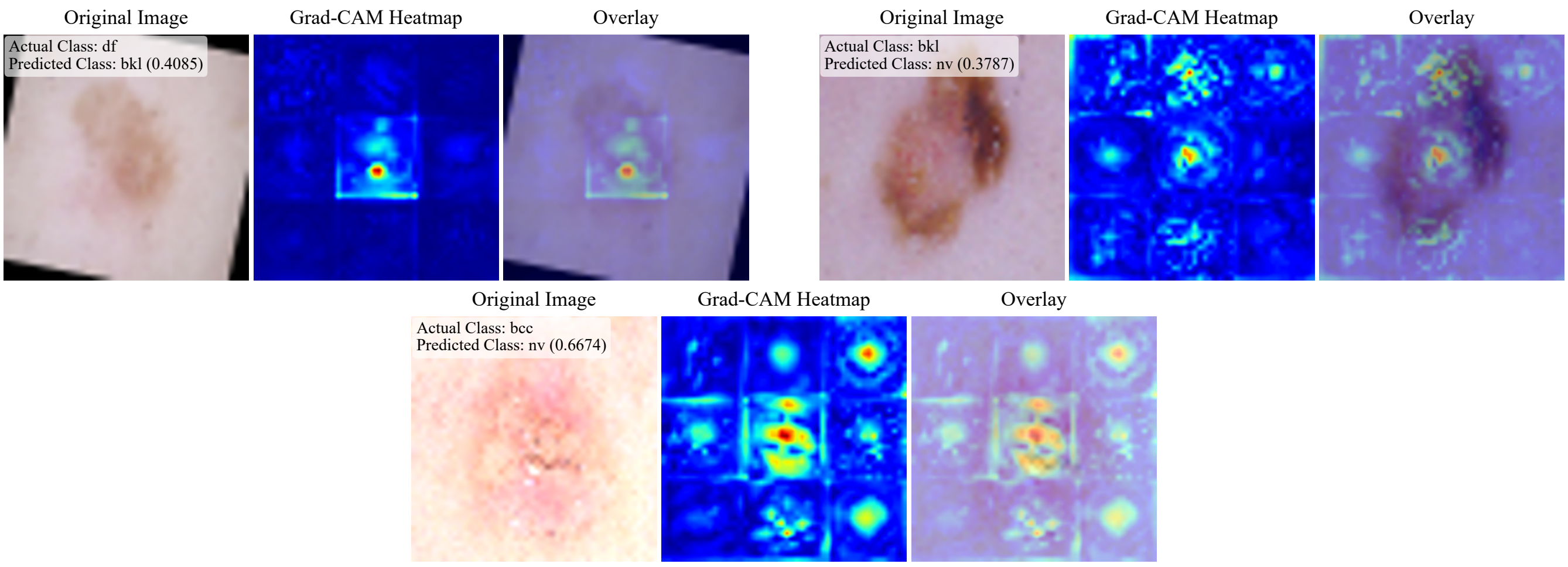

Figure S4. Additional Grad-CAM visual explanations for representative misclassified samples not shown in the main manuscript. From left to right and top to bottom: DF, BKL, and BCC. VASC is not included because all test samples were classified correctly.
